# Context‐dependent costs and benefits of tuberculosis resistance traits in a wild mammalian host

**DOI:** 10.1002/ece3.4699

**Published:** 2018-12-06

**Authors:** Hannah F. Tavalire, Brianna R. Beechler, Peter E. Buss, Erin E. Gorsich, Eileen G. Hoal, Nikki le Roex, Johannie M. Spaan, Robert S. Spaan, Paul D. van Helden, Vanessa O. Ezenwa, Anna E. Jolles

**Affiliations:** ^1^ Department of Integrative Biology Oregon State University Corvallis Oregon; ^2^ The Institute of Ecology and Evolution University of Oregon Eugene Oregon; ^3^ College of Veterinary Medicine Oregon State University Corvallis Oregon; ^4^ SANPARKS Skukuza South Africa; ^5^ South African Medical Research Council, DST/NRF Centre of Excellence for Biomedical TB Research, Division of Molecular Biology and Human Genetics, Faculty of Health Sciences Stellenbosch University Tygerberg South Africa; ^6^ Department of Fisheries and Wildlife Oregon State University Corvallis Oregon; ^7^ Odum School of Ecology and Department of Infectious Diseases, College of Veterinary Medicine University of Georgia Athens Georgia; ^8^ Present address: Prevention Science Institute University of Oregon Eugene Oregon; ^9^ Present address: Institute of Ecology and Evolution University of Oregon Eugene Oregon; ^10^ Present address: Erin E. Gorsich, Zeeman Institute: Systems Biology and Infectious Disease Epidemiology Research (SBIDER) University of Warwick Coventry UK; ^11^ Present address: School of Life Sciences University of Warwick Coventry UK

**Keywords:** African buffalo, coevolution, heritability, host‐pathogen, pace‐of‐life

## Abstract

Disease acts as a powerful driver of evolution in natural host populations, yet individuals in a population often vary in their susceptibility to infection. Energetic trade‐offs between immune and reproductive investment lead to the evolution of distinct life history strategies, driven by the relative fitness costs and benefits of resisting infection. However, examples quantifying the cost of resistance outside of the laboratory are rare. Here, we observe two distinct forms of resistance to bovine tuberculosis (bTB), an important zoonotic pathogen, in a free‐ranging African buffalo (*Syncerus caffer*) population. We characterize these phenotypes as “infection resistance,” in which hosts delay or prevent infection, and “proliferation resistance,” in which the host limits the spread of lesions caused by the pathogen after infection has occurred. We found weak evidence that infection resistance to bTB may be heritable in this buffalo population (*h* ^2^ = 0.10) and comes at the cost of reduced body condition and marginally reduced survival once infected, but also associates with an overall higher reproductive rate. Infection‐resistant animals thus appear to follow a “fast” pace‐of‐life syndrome, in that they reproduce more quickly but die upon infection. In contrast, proliferation resistance had no apparent costs and was associated with measures of positive host health—such as having a higher body condition and reproductive rate. This study quantifies striking phenotypic variation in pathogen resistance and provides evidence for a link between life history variation and a disease resistance trait in a wild mammalian host population.

## 1 INTRODUCTION

Disease resistance traits can evolve rapidly as a result of coevolution between hosts and pathogens (van Valen, 1973). Resistance traits provide fitness benefits to the host in the presence of the pathogen, but immune defenses required for resistance are often energetically costly or limited by physiological or genetic constraints of the host (Ardia, Parmentier, & Vogel, 2011; Downs, Adelman, & Demas, 2014). As a result, the host faces trade‐offs between disease resistance and other physiological processes, such as reproduction or growth (Boots & Haraguchi, 1999; Zuk & Stoehr, 2002). For example, trade‐offs in immunity and key physiological processes have been well studied in birds, linking infection and resulting immune activity to variation in molting ability (Marzal et al., 2013; Moreno‐Rueda, 2010), growth rate (Gallizzi, Alloitteau, Harrang, & Richner, 2008; Soler, Neve, Perez‐Contreras, & Soler, 2003), and reproductive investment (Allander, 1997; Oppliger, Christe, & Richner, 1997; Soler, Martin‐Vivaldi, Haussy, & Moller, 2007). Context‐dependent costs and benefits to the host can lead to frequency‐dependent selection dynamics in host populations dependent upon risk of infection, thus driving the evolutionary dynamics of resistance traits (Antonovics & Thrall, 1994; Boots & Haraguchi, 1999; Tellier & Brown, 2011).

The evolution of host disease resistance has been documented in multiple natural systems (Blanchet, Rey, & Loot, 2010; Bonneaud et al., 2011; Hasu, Benesh, & Valtonen, 2009; Hayward et al., 2011) and theoretical predictions that resistance traits should not reach fixation in host populations (Antonovics & Thrall, 1994; Best, White, & Boots, 2008) have largely been validated. This maintenance of variation in heritable resistance mechanisms suggests that, although resistance can confer advantages under strong selection imposed by the pathogen, resistance may not maximize fitness under all circumstances. If constitutively expressed resistance mechanisms that prevent infection come at a fitness cost, we would expect trade‐offs involving reduced fitness when the pathogen is absent (Boots & Haraguchi, 1999). Average lifetime fitness of the host may thus depend on the likelihood of infection, leading to ecological‐evolutionary feedbacks between disease dynamics and the frequency of heritable resistance traits in the host population (Boots & Haraguchi, 1999). Furthermore, since distinct resistance phenotypes likely arise from discrete underlying physiological mechanisms that carry unique fitness costs and benefits, multiple strategies could evolve within the same host population (Miller, White, & Boots, 2005; Restif & Koella, 2004). These resistance traits are not necessarily mutually exclusive and each individual occupies a phenotypic value along the continuum of each phenotype. This further complicates the study of coevolutionary dynamics within host populations, since selection acting on one resistance trait could affect the evolutionary trajectory of another (Ardia et al., 2011). Thus, it is important to consider multiple disease resistance strategies when testing hypotheses about mechanisms maintaining variation in disease resistance in natural populations.

Studies of laboratory and natural populations have revealed underlying genetic and immunological drivers of variation in resistance traits. For example, host genotype associates with variation in pathogen burden in many laboratory systems (Bruns, Carson, & May, 2012; Salvaudon, Heraudet, & Shykoff, 2007; Tavalire, Blouin, & Steinauer, 2016) and immune pathway knockout lines have demonstrated a direct relationship between immune function and overall pathogen burden in murine models (Grant et al., 2009; Kielian et al., 2007; Qiu et al., 2008). In wild populations of sheep, causative loci have been identified for strongyle (Beraldi et al., 2007) and nematode (Silva et al., 2012) resistance. Also, the rapid spread of *Mycoplasma* infection in American house finches has led to the discovery of the genetic and immune basis for resistance in this system (Adelman, Kirkpatrick, Grodio, & Hawley, 2013; Bonneaud et al., 2011). Though underlying casual variation in resistance traits has been characterized in multiple natural populations, few studies have tested hypotheses about the evolutionary mechanisms maintaining variation in these resistance traits (however, see Auld et al., 2013; Hayward et al., 2011; Hayward et al., 2014; Zhong, Pai, & Yan, 2005).

Resistance traits and associated costs are context‐dependent and often fluctuate with resource availability (Boots, 2011; Zuk & Stoehr, 2002), overall infection risk (Gandon & Vale, 2014), or the presence of coinfecting pathogens within the host (Mideo, Alizon, & Day, 2008). For example, a low resource diet led to higher levels of infection resistance in frogs challenged with gut nematodes, while higher resource availability favored worm tolerance (Knutie, Wilkinson, Wu, Ortega, & Rohr, 2017). Immune investment can also fluctuate with resource availability, as seen in tree lizards who are better able to balance the energetic demands of reproduction and wound healing when resources are plentiful, but reduce reproductive investment when resources are limited (French, Johnston, & Moore, 2007). In an experimental mouse‐nematode system, variation in overall infection risk led to a dose‐dependent increase in the production of pro‐inflammatory cytokines which negatively correlated with host fitness (Lippens, Guivier, Faivre, & Sorci, 2016). Coinfecting pathogens can also modify host immunity, leading to variation in resistance traits and associated costs. For example, intestinal helminth infection has been identified as a risk factor for both pulmonary tuberculosis (Elias, Mengistu, Akuffo, & Britton, 2006) and malaria (Druilhe, Tall, & Sokhna, 2005) infections in humans due to trade‐offs among branches of the immune system.

Multiple forms of disease resistance have been identified in the theoretical literature, often with disparate predicted fitness costs to the host due to intrinsic differences in underlying mechanisms (Best, White, & Boots, 2010; Boots & Bowers, 1999; Miller et al., 2005; Restif & Koella, 2004). “Infection resistance” is most commonly defined as the ability of a host to prevent infection by a pathogen (Simms & Triplett, 1994). For example, heritable variation in constitutively expressed innate pathogen recognition mechanisms could be energetically costly to the host, but prevent infection when the pathogen is present (Tellier & Brown, 2011; Zuk & Stoehr, 2002). Furthermore, in natural systems, differences among individuals in social behavior and habitat use can modify exposure risk (Hawley, Etienne, Ezenwa, & Jolles, 2011; Jolles, Ezenwa, Etienne, Turner, & Olff, 2008; Rushmore et al., 2013), resulting in heritable variation in infection, though behavioral mechanisms are commonly categorized as “avoidance” strategies (Boots & Bowers, 1999). Thus, variation in time to infection can arise from multiple, heritable mechanisms. Despite being commonly defined as a threshold trait, infection resistance likely operates on a continuum, with some animals succumbing to infection early in life, while others delay infection for longer periods of time. This variation in time to infection potentially leads to variation in costs and immune investment over the host's lifetime. Another form of resistance is “proliferation resistance” (previously also referred to as “control” in Miller et al., 2005). Proliferation resistance describes the host's ability to minimize the pathogen's growth rate once infected. Proliferation resistance is similar to disease tolerance because it potentially limits pathogen damage to the host, but unlike tolerance, proliferation resistance limits the growth rate of the pathogen, making these two host strategies evolutionarily distinct (Miller et al., 2005). In short‐lived infections, proliferation resistance corresponds to a high rate of pathogen clearance, while in chronic infections, proliferation resistance limits the growth rate of the pathogen within the host (e.g., limits spread across tissues or concentration of parasites in blood), but fails to eliminate the pathogen completely (Miller et al., 2005). Proliferation resistance likely arises from adaptive immune mechanisms involved in immune memory and pathogen containment (Keane et al., 1997; Mukhopadhyay et al., 2012; Sandler, Mentink‐Kane, Cheever, & Wynn, 2003), which may come at a lower energetic costs than constitutively expressed infection resistance mechanisms (Boven & Weissing, 2004; Goldszmid & Trinchieri, 2012). Proliferation resistance is also mechanistically distinct from tolerance, which lessens the pathogen's impact on host fitness through mechanisms of tissue repair or downregulation of pro‐inflammatory pathways (Medzhitov, Schneider, & Soares, 2012; Sears, Rohr, Allen, & Martin, 2011).

Varying resistance strategies can have disparate effects on life history evolution due to their context‐dependent costs and trade‐offs (Zuk & Stoehr, 2002). Trade‐offs in immune function, reproduction, and life span have been characterized within the context of “pace‐of‐life” life history syndromes (Stearns, 1989; Zera & Harshman, 2001). A “fast” pace‐of‐life is characterized by increased investment in constitutively expressed, innate immune defenses, early reproduction, and a shorter life span, while a “slow” pace‐of‐life is exemplified by the formation of adaptive immune memory, slower reproduction, and a longer life span (Martin, Hasselquist, & Wikelski, 2006). Putting disease resistance phenotypes into the context of these pace‐of‐life syndromes, we might expect infection‐resistant animals to exemplify a fast pace‐of‐life through constitutively expressed immune defenses, while proliferation resistant animals would exemplify a slow pace‐of‐life with induced pathogen clearance.

**Figure 1:**
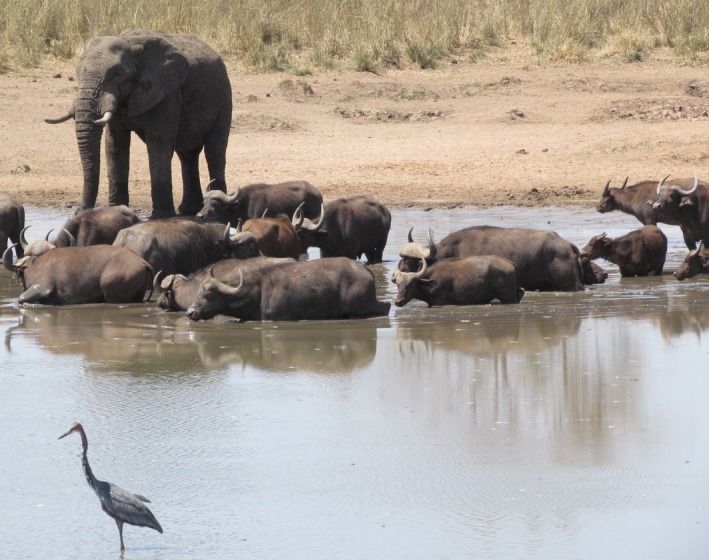
African buffalo (*Syncerus caffer*) serve as a maintenance host for bovine tuberculosis (bTB) in the savanna ecosystem. Seasonally limited resources force animals into close proximity, increasing the likelihood of disease spread

Here, we explore phenotypic variation in two forms of resistance to a globally important pathogen of livestock, wildlife, and people, *Mycobacterium bovis*, in a free‐living population of African buffalo (*Syncerus caffer*, Figure 1). *Mycobacterium bovis* is the causative agent of bovine tuberculosis (bTB) and a zoonotic bacterial pathogen with a broad host range, often leading to long‐term infection with high morbidity and eventual mortality in mammals (Ayele, Neill, Zinsstag, Weiss, & Pavlik, 2004; Rua‐Domenech, 2006; Welburn, Beange, Ducrotoy, & Okello, 2015). The host immune system forms granulomas (lesions) around infected tissue, often resulting in large areas of necrosis in the lungs and ultimately, death (Russell, 2007). bTB infection has been previously shown to reduce survival, pregnancy rates, and condition in African buffalo (Ezenwa & Jolles, 2015; Jolles, Cooper, & Levin, 2005). Given the negative fitness effects of bTB infection, we ask the following: (a) Do African buffalo vary in their ability to prevent infection or limit proliferation of *M. bovis* once infection occurs? (b) Is phenotypic variation in the host response to bTB heritable? And, (c) Are there fitness costs associated with infection or proliferation resistance to bTB? We used a longitudinal study in which 200 buffalo were captured every six months for 4 years to address these questions. We used age at bTB conversion as a continuous measure of infection resistance since there is no evidence that African buffalo clear infection before death (Bengis, 1999). Exposure and infection risk likely varied among buffalo; however, using age at first infection allowed us to assess what proportion of variation in infection resistance is due to heritable mechanisms (e.g., immunity, behavior). Additionally, within a subset of these animals that were culled at the end of the study, we use lung lesion count as a continuous measure of proliferation resistance, as it corresponds to immune containment of *M. bovis*.

## 2 METHODS

### 2.1 Study area and field data collection

Two hundred subadult and young adult female African buffalo (initial ages 2–7 years) were captured every six months in the southern part of Kruger National Park, South Africa between June 2008 and August 2012 as part of a longitudinal study of coinfection (for more detail, see Ezenwa & Jolles, 2015; Ezenwa & Jolles, 2011). These buffalo were sampled from two distinct herds occurring in the Crocodile Bridge and Lower Sabie areas of the park. The Crocodile Bridge herd included buffalo in the area around the Crocodile River in the southeast extent of the park, while north of this the Lower Sabie herd included buffalo near the eastern reaches of the Sabie River. The total population size for this area was estimated to be approximately 2,500 animals during the capture period (Ezenwa & Jolles, 2015).

Each buffalo was fitted with either a radio (*n* = 193) or a satellite (*n* = 7) collar with a high‐frequency VHF transmitter upon first capture, which was then used to locate them for subsequent captures. Individuals lost to death or emigration during the study period were replaced to maintain a constant sample size of approximately 200 animals spread equally between the two herds (*n*_total_ = 317). Of these animals, half (*n* = 50 per herd) were randomly chosen to receive an antihelminthic bolus (slow‐release fenbendazole [Panacur, Intervet]) as part of the study design outlined in Ezenwa and Jolles (2015). Previous work in this group of buffalo demonstrated that antihelminthic treatment does not affect the probability of bTB infection, but does increase the likelihood of survival following infection (Ezenwa & Jolles, 2015). We are therefore confident that treatment does not significantly influence observed infection resistance; however, we account for antihelminthic treatment in all models measuring postinfection fitness.

At each capture, animals were immobilized by dart from a helicopter or truck using etorphine (M99, Novartis, Kempton Park, South Africa; Captivon, Wildlife Pharmaceuticals, Karino, South Africa) and azaperone (Stresnil, Jansen Pharmaceuticals, Halfway House, South Africa). Following data collection, immobilization was reversed using diprenorphine (M5050, Novartis) and naltrexone (40 mg/ml, Kyron laboratories, Benrose, South Africa). Animals were kept under observation until recovered, and all immobilizations were conducted by a veterinarian according to the South African National Parks Standard Operating Procedures for the Capture, Transportation, and Maintenance in Holding Facilities of Wildlife. All animal work for this study was approved by the institutional animal care and use committee at Oregon State University (ACUP #3267) and the University of Georgia (UGA No. A201010190‐A1).

Age at each capture was determined in young animals by tooth emergence and in older animals by wear pattern per established methods in this species (Jolles, 2007). Pregnancy status and stage were determined by rectal palpation done by a wildlife veterinarian. This method shows 100% sensitivity in Egyptian buffalo (*Bos bubalis*) after 51 days of gestation (Karen et al., 2011) and has been validated in this study herd (Beechler et al., 2017). The presence of a calf at heel was detected visually or by evidence of lactation (manual milking of all four teats; Jolles et al., 2005), and calves were aged by body size and horn shape. Due to differences in total observation period (time in study), reproductive rate was used as a proxy for fitness instead of the total number of calves per individual. Reproductive rate was calculated by dividing the total number of calves per individual by the number of years that individual was observed to obtain an estimate of calves per year.

Body condition was assessed on a five‐point scale through palpation and visual inspection of four areas where buffalo deposit fat: spine, hips, ribs, and base of tail. Condition ranged from 1 (very poor) to 5 (excellent) at each area and was then averaged across these areas for an overall estimate of condition (Ezenwa, Jolles, & O'Brien, 2009). This method has been shown to correlate with fat deposits in the kidney (Ezenwa et al., 2009).

All blood samples for disease diagnostics were collected within fifteen minutes of a buffalo becoming immobilized and held on ice until analyzed to determine the animal's bTB status. bTB status was determined using a commercially available whole‐blood gamma interferon (IFNγ) assay (BOVIGAM, Prionics, Switzerland). This assay measures the difference in IFNγ production of whole blood in response to incubation with bovine versus avian tuberculin antigens, while controlling for differences in background IFNγ levels. Individual samples were called as bTB positive or negative based on absorbance thresholds optimized for bTB infection in African buffalo (Michel, Cooper, Jooste, Klerk, & Jolles, 2011). We obtained a time series of 2–9 bTB tests for each animal and used the full time series to more confidently assign bTB status. Animals with at least two consecutively positive bTB tests were assigned as bTB positive. Since bTB is chronic in buffalo and there is no evidence of recovery once infected, we assumed animals remain positive until death (Bengis, 1999). Animals with alternating test results or short observation periods (<3 captures) were not included in this work due to low confidence of phenotypic assignment. Additionally, since we evaluate reproductive rate as a metric of fitness relative to resistance traits and because body condition could be influenced by different energetic demands in juvenile and adult African buffalo, we included only those animals that had reached reproductive maturity before death or the end of the study in our analyses (four years of age (Carmichael, Patterson, Drager, & Breton, 1977); *n* = 190). We calculated age‐specific incidence of bTB infection as the proportion of animals that converted at each age (new cases) over the total number of animals at risk in that age group in the study population. We calculated age‐specific incidence for animals between two and eight years of age but lacked sufficient sample size of buffalo outside of this age range.

Although bTB was introduced into Southern Africa with European cattle, at this time, African buffalo serve as a maintenance host of bTB in the region, sustaining a relatively high prevalence (up to 27%) of *M. bovis* in some areas and facilitating infection of other hosts in the savanna ecosystem (Cross et al., 2009; Rodwell et al., 2001). bTB is most commonly transmitted through inhalation, colonizing lung and associated lymph tissues by infecting resident macrophages of the host (Kaufmann, 1991; Kornfeld, Mancino, & Colizzi, 1999; Raja, 2004). To evaluate pathology associated with bTB infection, a subset of bTB positive and negative animals were culled at the completion of the study (*n*_pos_ = 78, *n*_neg_ = 57). These animals were necropsied by experienced veterinarians and counts were taken of all tuberculosis‐associated lesions in the lungs and lymph tissue to assess differences in disease‐related pathology. Here, we use a subset of 33 of these culled animals for which age at conversion was known, to characterize proliferation resistance (all other bTB‐positive animals had converted at an unknown time before capture and we could not accurately assess time with bTB relative to the development of lesions).

### 2.2 SNP genotyping and filtering

We used single nucleotide polymorphism (SNP) based molecular methods to assess genetic differentiation among buffalo herds and to calculate pairwise estimates of relatedness for the heritability of infection resistance analysis, below. We extracted 100–200 ng genomic DNA from dried ear tissue samples (DNeasy blood & tissue kit, Qiagen) and prepared individual libraries for sequencing using type IIB restriction associated DNA (2bRAD) methods, detailed in Wang et al. (2012). Briefly, this method uses a type IIB restriction endonuclease (AlfI; Thermo Scientific #ER1801) to extract thousands of 36 bp reads from across the genome. We prepared genotyping libraries using reduced tag representation (RTR) as described in Wang et al. (2012) by ligating adaptors with 3′ overhangs ending in NC and NG. Samples were sequenced on an Illumina HiSeq 3000 sequencer at the Oregon State University Center for Genome Research and Biocomputing. We excluded terminal tag positions, ambiguous base calls, long homopolymer regions, and excessively low‐quality reads (>5 positions with quality <10). After trimming, the remaining high‐quality reads were retained for all subsequent mapping and genotyping. We extracted all AlfI cut sites (*n* = 480,162) from the *Syncerus caffer* genome (Glanzmann et al., 2016). We then used SHRiMP to map each individual sample to these loci and filtered the resulting matches for statistically weak or ambiguous alignments using parameters similar to those described by the software authors (Rumble et al., 2009). We determined genotypes at each AlfI site with >10× coverage, then filtered out any monomorphic loci. We allowed for 10% missing data at any given locus and one polymorphism per tag. Animals that were genotyped at 5,000 or fewer loci were removed from the dataset. We extracted scaffold and position information for each SNP for population structure analysis. The analysis pipeline outlined above was developed by Eli Meyer (available at https://github.com/Eli-Meyer). Markers were discarded if they were not biallelic, violated Hardy–Weinberg Equilibrium (*p* < 0.0001), or had a minor allele frequency less than 5%. Quality filtering yielded 187 usable samples genotyped at 1999 SNPs.

### 2.3 SNP‐based population structure

To test whether the two herds sampled were genetically distinct, we calculated global *F*_ST_ using filtered markers in the R packages *hierfstat* (Goudet, 2005). We observed a global *F*_ST_ value of 0.0003, leading us to conclude that these herds are not genetically distinct. This result agrees with previously reported behavioral observations of frequent herd switching and long‐distance dispersal in African buffalo (Caron, Cornelis, Foggin, Hofmeyr, & Garine‐Wichatitsky, 2016; Halley, Vandewalle, Mari, & Taolo, 2002; Naidoo, Preez, Stuart‐Hill, Beytell, & Taylor, 2014) and the lack of population differentiation observed in previous work in these herds using microsatellite markers (Lane‐deGraaf et al., 2015). We therefore consider any effect of “herd” in subsequent analyses as environmental and not reflective of differences in underlying genetic structure.

### 2.4 Variation in resistance

We quantified two types of bTB resistance in the African buffalo. First, infection resistance describes differences in time to onset of infection (i.e., conversion age), where animals that became bTB positive later in life or never converted are considered to be more infection resistant. We acknowledge that differences in age at conversion as we measured it here may reflect variation in underlying physiological mechanisms, but could also be due to heterogeneity in exposure among animals due to behavioral mechanisms. Here, we are interested in determining what proportion of variation in infection resistance is heritable, regardless of the causative mechanisms. Second, proliferation resistance describes differences in lung pathology relative to time since each animal first tested positive for bTB. We evaluated resistance traits in buffalo that were observed for at least 36 months and acquired bTB during the study period (*n* = 33). Infection resistance is a continuous trait, measured as age at bTB infection.

Lung pathology was assessed by removing the lungs and trachea from the buffalo and carefully palpating and visually examining for gross lesions (granulomas) by evaluating each lung lobe independently. If a lesion was noted, it was measured to describe the extent of lung area affected. The number of affected lung lobes was then tabulated, in addition to number, appearance, and area occupied by lesions. Here, we use total lung lesion count as a proxy for total pathology and a continuous measure of proliferation resistance. The formation of independent lesions directly corresponds to efficiency of immune containment mechanisms and control of bacterial growth rate in pulmonary tuberculosis, with fewer lesions indicating a more contained infection (Lin et al., 2014; Saunders & Cooper, 2000). Furthermore, lung lesion count was highly correlated with total lung area affected in these buffalo (*r* = 0.611; *p* = 0.0002) and outliers in area affected also had the highest lesion counts. Number of lesions was regressed onto the time since onset of infection to obtain a residual value relative to the expected average pathology given the length of infection using a generalized linear model with a Poisson error distribution. Those animals with positive residuals had more pathology than would be predicted for the time since onset of bTB, and are considered more proliferation susceptible, while those animals with negative residuals relative to the regression line had lower pathology than expected, and are therefore more proliferation resistant. To assess lymphatic infection, lymph nodes were bilaterally excised from the head (submandibular, tonsils, retropharyngeal, and parotid), thorax (bronchial and mediastinal), and periphery (axillary and prescapular). Each lymph node was sectioned by scalpel into 2–3 mm sections and the cut surface of each slice was evaluated for pathology as follows: nodes were marked as “positive” if they contained obvious, sometimes granular lesions containing purulent material, “suspect” if any focal firmness or density was present containing small pockets of purulent material, or “negative” if there were no indications of mature or early lesions. Analyses using the number of lymph nodes affected give similar results, and correlate tightly with lung lesions counts (*r* = 0.70; *p* < 0.0001).

To determine whether the two resistance phenotypes were correlated, we assessed the linear association between the time to onset of bTB (infection resistance) and the lesion‐time since infection residuals (proliferation resistance) using a Pearson's correlation.

### 2.5 Relatedness and heritability of resistance

We determined pairwise relatedness (r) among buffalo using the R package *related* (Pew, Muir, Wang, & Frasier, 2015) and the identity by decent‐based (IBD) estimator calculated in the maximum likelihood method of Milligan et al. (2003). To estimate the heritability of time to onset of bTB in this population, we used a mixed effects Cox model implemented within the R package *coxme* (Therneau, 2018). Animals that remained bTB negative during the study period were right‐censored in the model at their final age during the study period. The full model included treatment and final observed age as fixed effects and the final model was selected from all possible reduced models based on diagnostic checks for heteroscedasticity (residual plots) and Akaike's information criterion (AIC (Gurka, 2006); See Supporting Information Table S1). We estimated heritability within a time‐to‐event model using established methods that account for the proportion of censored observations in the dataset (Schneider, Strandberg, Ducrocq, & Roth, 2005; Yazdi, Visscher, Ducrocq, & Thompson, 2002). The following equation was used to estimate narrow sense heritability:

$$h_{\text{cen}}^{2}=\frac{V_{A}}{V_{A}+V_{E}+\frac{1}{1-c}}$$

Where *V*_A_ is the additive genetic variance, *V*_E_ is the environmental variance, and *c* is the proportion of observations requiring censoring in the model (here, *c* = 106/162 = 0.654). This correction method has been previously applied to human and livestock datasets (Anderson, Duffy, Martin, & Visscher, 2007; Schneider et al., 2005). We estimated the variance components *V*_A_ and *V*_E_ by incorporating both an IBD‐based relatedness matrix and a shared environmental (herd) matrix as the correlation structure of the random effect in our model. Using a likelihood ratio test in the R package *lmtest* (Zeileis & Hothorn, 2002), we compared the fit of the final model with and without relatedness or herd sharing matrices to determine if partitioning variance according to relatedness, herd structure, or both significantly improved the fit of the final model. This test allowed us to assess the significance of our variance components and the heritability estimate for this trait. Here, we also report the uncensored heritability estimate in Table 1 as an upper limit of the true value, and to allow for the estimation of standard error. We estimated standard error for both estimates using the “h2G” function in the R package *gap* (Zhao, 2007). We assigned the standard error of the uncensored estimate to both the censored and uncensored estimates of heritability, as no method for censored standard error calculation has been described (Schneider et al., 2005). We lacked a sufficient sample size of culled animals to estimate the heritability of proliferation resistance in this population, as only three pairs within 33 culled animals contained relatives in the dataset (*r* > 0).

**Table 1:** Heritability of infection resistance

Model	Estimate (*SE*)[Link]	*p* value*
Risk of bTB onset	—	0.003
Treatment (control)[Link]	1.013	0.960
Variance components		
*V*_A_	0.329 (0.045)	
*V*_E_	0.207 (0.036)	
*h* ^2^ =	**0.615** (0.573)	
$h_{\text{cen}}^{2}$ =	**0.096** [Link] (0.573)	

Fixed effect estimates are natural log back‐transformed and represent a multiplicative increase in risk of bTB.

Treatment refers to buffalo that were not treated for worms (control) versus buffalo that were treated for worms (antihelminthic bolus; reference group).

The censored heritability estimate is corrected for the proportion of observations censored in the analysis (Schneider et al., 2005).

a *p* values were estimated using a Cox mixed effects model.

### 2.6 Cost of resistance

To evaluate the fitness costs of bTB resistance in culled, converted buffalo (*n* = 33), we averaged body condition and calculated reproductive rate across the observation periods before and after bTB conversion for each culled animal. We then used linear mixed effects models in the R package *nlme* (Pinheiro, Bates, DebRoy, Sarkar, & R Core Team, 2018) to measure the effect of each resistance phenotype on these fitness measures. We used age at conversion and lung pathology residuals as continuous metrics of infection‐ and proliferation resistance, respectively. The full mixed effects model for each fitness metric included time (“before” and “after” conversion), herd, antihelminthic treatment, age at first capture, the two continuous resistance metrics, and animal ID as a random effect. Initially, interaction terms for both resistance phenotypes and an interaction of each resistance phenotype with time were included in each full model, but none were retained following model selection. Model selection involved the comparison of all possible reduced models and was based on marginal *R* ^2^, diagnostic checks for heteroscedasticity (residual plots), and AIC (Gurka, 2006; Supporting Information Table S2).

To assess the survival costs of infection resistance, we compared postconversion survival times of all bTB‐converted animals (*n* = 56) using a Cox proportional hazards survival analysis in the R package *survival* (Therneau, 2015). We selected the final model for these data using AIC and *R* ^2^ values (Supporting Information Table S3). The final model contained conversion age as a continuous metric of infection resistance, as well as herd and antihelminthic treatment, factors which have previously been shown to impact survival following bTB infection in this group of buffalo (Ezenwa & Jolles, 2015). All analyses were run in R version 3.2.4 (R Core Team, 2016).

## 3 RESULTS

### 3.1 Variation in host response to bTB

At the beginning of the study, prevalence of bTB infection was 0.142 and did not differ significantly among the two herds (*n* = 176, *χ* ^2^ = 0.987, *p* = 0.320) or antihelminthic treatment groups (*χ* ^2^ = 0, *p* = 1.0). Within our sample (*n* = 187), 25 buffalo were initially bTB positive, 106 remained bTB negative, and 56 acquired bTB infection during the study period.

**Figure 2:**
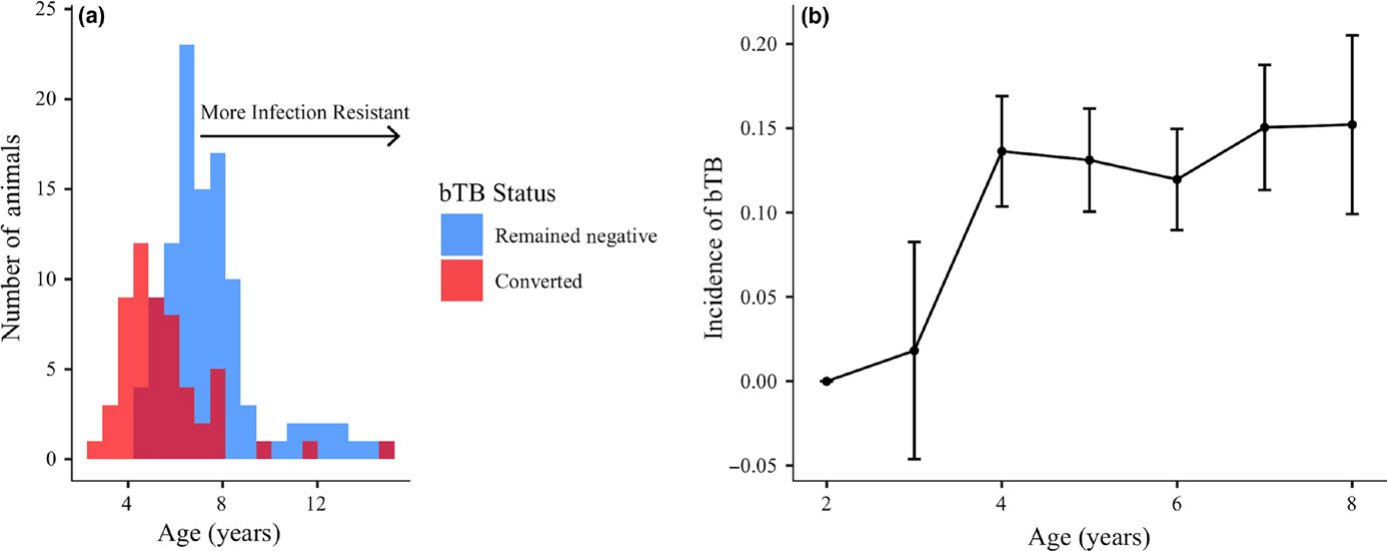
Bovine tuberculosis infection patterns in African buffalo. (a) Conversion age distribution of animals that converted to bovine tuberculosis (bTB) positive during the study period (red, *n* = 56) and the final observed age distribution of animals that remained bTB negative throughout the study period (blue, *n* = 106). The dark red area represents the overlap in the two histograms. Animals that converted later in life or remained bTB negative to a later age are considered more infection resistant than those that converted at a young age. Animals that were bTB positive at first capture are not shown (*n* = 26). (b) Observed age‐specific incidence of bTB calculated as the number of animals that converted to bTB positive (new cases) over the total number at risk for each age. Incidence of bTB increased to age four, then leveled off and remained relatively constant in this herd of buffalo (*n* = 202). Error bars represent standard error

**Figure 3:**
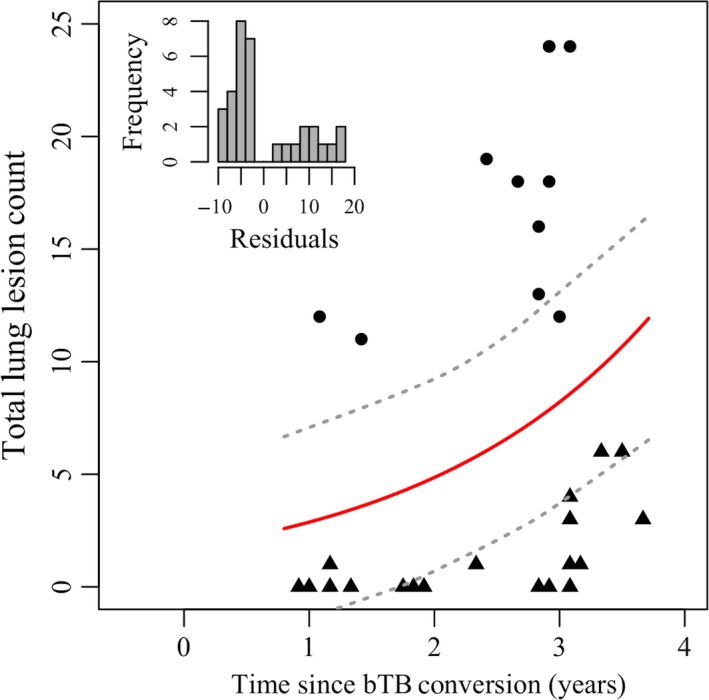
Lung pathology associated with bovine tuberculosis infection reflects level of proliferation resistance. The graph displays the number of bovine tuberculosis (bTB) lung lesions over time since conversion to bTB positive. Here we see two distinct groups of animals on either side of the nonlinear prediction line (red with gray 95% confidence bands; *y* = 1.7 × 1.69*^x^*). Those observations above the regression line had positive residuals and are considered proliferation susceptible (circles), and had a higher number of lesions than would be expected given time since conversion. Those points below the regression line had negative residuals and are considered proliferation resistant (triangles) and had less pathology than predicted. The inset histogram of residual values displays these two distinct groups

**Figure 4:**
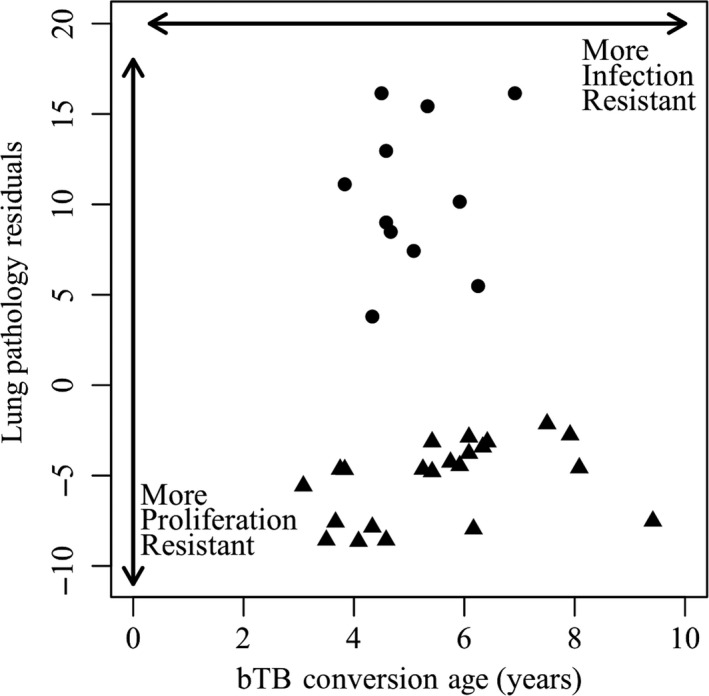
Individuals within resistance trait space. We observed high variation in both time to onset of infection (infection resistance) and lung lesions accumulated over time (proliferation resistance‐ triangles denote low lesion accumulation). The two resistance phenotypes were not correlated, with some African buffalo displaying high values of one form of resistance, some displaying high values of both forms of resistance, and others displaying neither form. Each converted, culled animal is represented one time along each continuous axis in resistance trait space (*n* = 33)

Conversion age varied broadly, from two to greater than ten years of age (Figure 2a). The age‐specific incidence increased until age four and then remained relatively constant (Figure 2b). Mean age at conversion during the study period was 5.5 years (*n* = 56 of 187; 95% CI (4.980, 6.060)); and a subset of animals remained bTB‐negative throughout the study period, many of which exceeded 5.5 years of age (*n* = 93 of 106). Among bTB‐infected animals, the number of lung lesions generally increased with time since infection, although almost a third of culled animals remained lesion‐free in their lungs despite a positive bTB test. We found high variation among animals in proliferation resistance, with some animals having fewer total lesions than predicted by the lesion number over time since onset nonlinear regression and others developing lesions much faster (Figure 3). Interestingly, we observed striking variation in lesion number on either side of our prediction line, with animals having greater than ten lesions or less than six, regardless of time since conversion. Age at conversion (infection resistance) and pathology regression residuals (proliferation resistance) were not correlated (*n* = 33, *r* = −0.051, *p* = 0.780) as apparent by the spread of points in Figure 4 along the two continuous axes of resistance (“resistance trait space”), indicating that these resistance traits likely arise from distinct underlying mechanisms.

### 3.2 Heritability of host resistance

We used marker‐based relatedness estimates within a censored time‐to‐event variance‐partitioning model to estimate heritability of infection resistance in this herd of African buffalo. These data present a unique problem for the estimation of heritability for the time to onset of bTB infection as we observed few closely related pairs by chance within our subset (we sampled ~8% of the total population, yielding 21 half‐sibling pairs within 187 animals). We removed any animals that were initially bTB positive from this analysis, since their conversion age is unknown (*n* = 25). After this filtering, our final dataset for estimating the heritability of infection resistance included 162 African buffalo. Due to a limited sample size within the culled subset, we lacked power to estimate the heritability of proliferation resistance in this herd. We obtained a low censored heritability estimate of 0.095 for infection resistance as measured by bTB conversion age in this population of buffalo (Table 1). Due to low bTB prevalence in the herd leading to a high proportion of right‐censored observations in this sample (i.e., 66% of animals did not become infected during the observation period), we predict this is a gross underestimation of the true heritability of this trait. The uncensored heritability estimate of 0.615 (*SE* = 0.573) likely represents the upper limit for the trait heritability, while the censored estimate serves as the lower limit. Using a likelihood ratio test for goodness of fit, we found that including the relatedness matrix as the correlation structure of the random effect in the heritability model did not significantly improve the fit of the final model (ΔLL = −179.55, *χ* ^2^ = 359.09, *p* < 0.001), but including the herd sharing matrix did (ΔLL = 6.65, *χ* ^2^ = 13.29, *p* = 0.010). These results suggest that heritable factors are not significantly contributing to variation in time to onset of bTB in this sample, but that herd membership significantly predicts variation in time to onset of bTB.

### 3.3 Fitness costs and benefits of resistance phenotypes

**Figure 5:**
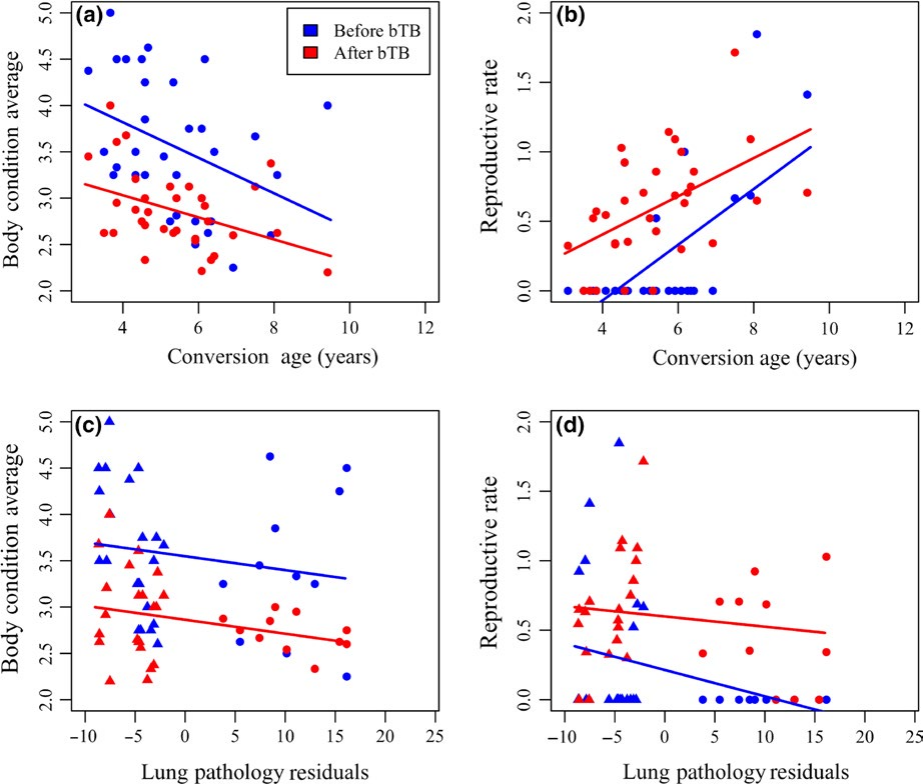
Fitness costs of each resistance trait. Fitness costs of resistance before (blue) and after (red) bTB infection in converted, culled buffalo (*n* = 33). Body condition and reproductive rate (calves/year) were assessed in each animal relative to a continuous metric of infection resistance (conversion age; a–b) and a continuous metric of proliferation resistance (pathology residuals in the lung lesion count over time since onset nonlinear regression; c–d). Triangles denote negative residuals and more proliferation resistant animals, while circles denote positive residuals and less proliferation resistant animals. Both resistance traits were included in each fitness model, however, here resistance traits are graphed separately to show main effects on fitness measures. Overall, more infection‐resistant animals (later conversion age) had lower body condition but a higher reproductive rate, while more proliferation resistant animals (lower pathology) had a higher body condition and reproductive rate. Each converted, culled animal is represented twice in each fitness graph with an average before‐ and after bTB infection fitness measurement

In order to compare costs associated with each resistance strategy, we compared continuous variation in infection and proliferation resistance among animals that acquired bTB infection during the study to three metrics of buffalo fitness before and after conversion: reproductive rate, body condition, and survival. We observed an overall decrease in body condition due to bTB infection and an increase in reproductive rate over time (*n* = 33; Table 2, Figure 5). These overall trends were expected given that all animals in this analysis became bTB positive, which has been previously shown to reduce body condition (Ezenwa & Jolles, 2015; Jolles et al., 2005), and many of the young females we sampled reached reproductive age during the study. To control for age‐dependent differences in reproductive rate, we retained age at first capture in the final model of this fitness metric. We observed a cost of infection resistance in the form of lower body condition and reduced survival time following infection. For each year increase in conversion age, animals lost body condition but had an overall higher reproductive rate (Table 2, Figure 5a,b). Due to the age‐dependency of our infection resistance classification, comparing overall survival time (final age at death or last observation) relative to conversion age was not meaningful. However, we found infection‐resistant animals had marginally lower survival following infection than those that succumbed to infection earlier in life, as we observed a 1.298 fold increased risk of death per year increase in conversion age (Table 3, *p* = 0.078). We observed no reproductive or body condition costs associated with proliferation resistance (Table 2, Figure 5c,d). Decreasing lung pathology was associated with increased body condition and calving rate both before and after disease onset.

**Table 2:** Measures of health and fitness before and after bovine tuberculosis (bTB) infection

Model	Estimate (*SE*)[Link]	*t*‐value	*p* value*
A. Average body condition (*n* = 33)	3.936 (0.263)	14.965	<0.001
Time (after bTB)	−0.685 (0.116)	−5.914	<0.001
Conversion age (years)	−0.131 (0.042)	−3.127	0.004
Lung pathology residuals	−0.018 (0.007)	−2.358	0.025
Herd (Lower Sabie)	0.528 (0.123)	4.288	<0.001
B. Average reproductive rate (*n* = 33)[Link]	−0.784 (0.192)	−4.088	<0.001
Time (After bTB)	0.385 (0.082)	4.693	<0.001
Conversion age (years)	0.188 (0.053)	3.562	0.001
Lung pathology residuals	−0.014 (0.005)	−2.612	0.015
Treatment (bolus)	−0.126 (0.092)	−1.374	0.181
Herd (Lower Sabie)	0.183 (0.087)	2.117	0.044
Age at first capture	−0.012 (0.057)	−0.022	0.829

Estimates for each multi‐level factor are interpreted as the difference relative to the reference level, given the factor level in parentheses.

The model for reproductive fitness (B) includes years in study as an offset term to control for differences in observation period.

a *p* values were estimated using a linear mixed effects model fit by maximum likelihood.

**Table 3:** Death risk following bovine tuberculosis (bTB) infection

Model	Estimate (95% CI)[Link]		*p* value*
Death risk (*n* = 56, events = 11)			
Conversion age (years)	1.298	(0.971, 1.736)	0.078
Treatment (bolus)	0.135	(0.025, 0.715)	0.019
Herd (Lower Sabie)	7.179	(0.567, 90.874)	0.128
			Adjusted *R* ^2^ = 0.176

Estimates are natural log back‐transformed and represent a multiplicative increase in risk of death per unit increase in each predictor.

a *p* values were estimated using a Cox proportional hazard model.

## 4 DISCUSSION

Here, we identify two distinct forms of disease resistance in a natural host–pathogen system, which vary in their relative fitness costs and benefits. We observed clear costs of infection resistance in the form of reduced condition and marginally reduced survival once infected in animals that converted to bTB positive later in life. In addition to our survival observations, poor body condition has been previously shown in this system to be a strong predictor of mortality (Budischak, O'Neal, Jolles, & Ezenwa, 2018; Gorsich, Ezenwa, Cross, Bengis, & Jolles, 2015). Therefore, the infection resistance phenotype appears to be advantageous only in terms of preventing or delaying bTB infection, but carries a survival cost if bTB does occur. Additionally, if underlying mechanisms of infection resistance are costly, this resistance trait is likely also associated with lower survival even in the absence of bTB, as evidenced by decreased body condition. On the other hand, infection resistant buffalo had a higher reproductive rate than animals that became bTB‐infected earlier in life, even after controlling for age‐dependent differences in reproductive rate. Taken together, these findings point to an association between (a) a faster pace‐of‐life in infection resistant buffalo (higher reproductive rates before infection), and (b) improved resistance to infection, but poor survival with bTB. Additionally, as reported previously in this herd (Ezenwa & Jolles, 2015), we observed a dramatic reduction in risk of death following bTB infection in those animals that received an antihelminthic bolus. This could potentially modify the observed fitness advantages and costs of both resistance traits through immunomodulation or reduced competition for resources within the host (Ezenwa & Jolles, 2011); however, we lack the data to address these questions here. To our knowledge, this is one of few studies providing direct evidence for a cost of resistance in a natural animal system (but see Auld et al., 2013; Zhong et al., 2005; Bonneaud, Balenger, Hill, & Russell, 2012; Graham et al., 2010; extensive examples in plant systems are reviewed in Brown & Rant, 2013; Burdon & Thrall, 2003; Meyers, Kaushik, & Nandety, 2005). Animals higher on the continuum of proliferation resistance (negative residuals) had better condition and a higher reproductive rate, suggesting that this resistance phenotype could be a trait of animals in generally better health or those with more energetic resources.

Pathogen virulence has been shown to drive the evolution and maintenance of resistance phenotypes in many wild systems (Ferrandon, 2009; Little, Shuker, Colegrave, Day, & Graham, 2010). Theory suggests that for infection resistance to evolve in a system, pathogen virulence must be high such that the costs of resisting are less than the negative fitness effects of succumbing to infection over the lifetime of the animal (Boots & Haraguchi, 1999). Since body condition is highly predictive of survival in buffalo, energetically costly infection resistance mechanisms, constitutively expressed regardless of infection risk, may confer negative fitness effects in the absence of bTB. However, these immune mechanisms, if general enough, could confer resistance to other pathogens as well, complicating the evolutionary dynamics of this trait depending on the relative virulence of endemic pathogens. Furthermore, if bTB force of infection is high, and most animals get infected before reproductive age, we would expect strong directional selection for infection resistance in this system. Conversely, if bTB force of infection is low and infection resistance mechanisms do not confer resistance to other virulent pathogens, we would expect the condition costs of infection resistance to impact long‐term reproductive rates and survival, resulting in directional selection away from this trait. Interestingly, bTB was only recently detected in the African buffalo of Kruger National Park in 1990 (Rodwell et al., 2001), therefore representing a “novel” pathogen and coevolutionary partner relative to other endemic pathogens in the region including Rift Valley fever (Beechler, et al., 2015), brucellosis (Gorsich et al., 2015), and schistosomes (Beechler et al., 2017). The high phenotypic variation observed in these resistance traits could also result from plasticity or other physiological or environmental factors not addressed in this study. However, here, we detect significant differences in fitness metrics relative to measures of infection and proliferation resistance, suggesting that the resistance strategies described here carry fitness benefits and costs and may have evolved over a relatively short time evolutionarily. We did not detect heritable variation in either trait, though we did observe a nonzero estimate of heritability in this sample. This could be largely due to a small sample size and the nature of our measure of infection resistance. Since infection resistance is an age‐based trait, and we only observed these animals for four years, it is likely that the age at last observation was an inaccurate estimate of true conversion age is this censored time to event analysis. Also, we observed very few related individuals in this subset of buffalo, suggesting that a larger sample of this population would lead to a more accurate estimate of trait heritability. Thus, in order to assess the true fitness advantages and resulting evolutionary dynamics of these resistance phenotypes, we would need to quantify lifetime reproductive success in a larger proportion of the total population and evaluate these resistance traits within the context of potential coinfecting pathogens.

Trade‐offs leading to the maintenance of variation in disease resistance in wild populations can be interpreted within the context of life history theory. Specifically, difference in pace‐of‐life syndrome involving trade‐offs between reproductive and immune investment (Sears et al., 2011). Having identified two continuous resistance traits that appear to vary in reproductive and life span strategies, we propose a difference in life history syndrome among individuals displaying extreme values of each resistance type. Several studies in recent years have invoked life history theory to explain relative investment in constitutively expressed, general immunity versus inducible long‐term immune memory (Ardia et al., 2011; Miller, White, & Boots, 2007; Previtali et al., 2012; Sandmeier & Tracy, 2014). These ideas have been explored theoretically (Boots, Donnelly, & White, 2013), as well as in some natural systems (e.g., amphibians: Johnson et al., 2012, birds: Hasselquist, 2007; Jacques‐Hamilton et al., 2017; Tieleman, Williams, Ricklefs, & Klasing, 2005, rodents: Previtali et al., 2012; Rynkiewicz et al., 2013, and sheep: Graham et al., 2010). Specifically, organisms following a “fast” pace‐of‐life invest disproportionately in constitutively expressed, general immune mechanisms, and reproduce earlier in life, while organisms exemplifying a “slow” pace‐of‐life invest in immune memory that will protect them throughout a longer life span (Previtali et al., 2012). Though the constitutively expressed immune response associated with a “fast” pace‐of‐life may offer immediate protection from ubiquitous exposure to pathogens, innate immune mechanisms often produce severe collateral damage to host tissues that is costly to mitigate (Goldszmid & Trinchieri, 2012). Additionally, energetic costs of constitutive immune protection are present even when the pathogen is not. Here, we see evidence of a “fast” life history strategy in infection resistant buffalo, as they experience a significant loss in condition (a proxy for energy reserves; see Figure 5), but invest more in reproduction preinfection. A reduction in condition before infection may suggest increased resource allocation to constitutively expressed immune mechanisms or repair of collateral damage, resulting in the delay or prevention of bTB infection, although we did not directly assess specific mechanisms of immunity here. Furthermore, infection resistant buffalo that converted to bTB later in life had an overall higher reproductive rate over the study period than animals that converted earlier, suggesting a reproductive advantage of delaying infection. However, the fitness advantages of infection resistance cease upon infection, as these animals are at higher risk of death once infected. Taken together, these findings provide evidence for the disproportionate investment in fitness before infection exemplary of a “fast” pace‐of‐life. Here, we provide strong evidence of interindividual variation in life history strategy among individuals in a single population.

In contrast, we observed no fitness costs associated with higher levels of proliferation resistance, suggesting that this resistance phenotype associates with potentially higher energy stores and higher reproductive fitness in this population. This finding implies that at the onset of bTB, animals with higher initial fitness and condition suffer less pathology or can more effectively mitigate damage than animals in poor condition, which may lead to overall higher survival rates in this group, since condition is highly predictive of survival in buffalo (Budischak et al., 2018; Gorsich et al., 2015). It has been demonstrated across taxa that organisms in better condition have higher available resources to allocate to immune coping mechanisms or tolerate resource leaching by the pathogens themselves, especially in environments where resources are seasonally limited (Martin, Weil, & Nelson, 2008). Furthermore, reproductively mature ruminants often prioritize energetic allocation to growth, pregnancy, and lactation over immune function when resources are limited (Coop & Kyriazakis, 1999), suggesting that buffalo of lower condition could be allocating fewer resources to proliferation resistance once infected. Unfortunately, because these animals were culled, we cannot directly address a survival advantage of proliferation resistance. We also could not estimate heritability of this trait in the current study due to low sample size of the culled population, so we could not estimate the genetic contribution to the proliferation resistance phenotype.

We also conclude that the two resistance phenotypes are not correlated in this population, suggesting that they arise from distinct physiological mechanisms. This is to be expected due to differences in the nature of each defense strategy: infection resistance likely results from strong pathogen recognition or pathogen clearing, while variation in proliferation resistance likely arises from differences in pathogen containment strategies or degradation (e.g., granuloma formation in the lungs: Russell, 2007). Distinct, noncorrelated forms of resistance to *M. tuberculosis* have also been observed in humans, as differentiated by reaction to the Tuberculin Skin Test (TST). Variation in TST has been linked to two distinct genomic regions: one explaining variation in overall resistance to *M. tuberculosis* infection, and another explaining variation in the severity of the TST response in positive individuals (Cobat et al., 2009). Additionally, variation in distinct forms of pathogen defense, dependent on host genetic background, has been demonstrated in other disease systems (e.g., snail‐schistosomes: Tavalire et al., 2016, *Daphnia*‐*Pasteuria*: Vale & Little, 2012, mouse‐malaria: Raberg, Sim, & Read, 2007), though rarely outside of the laboratory (but see Beraldi et al., 2007; Hayward et al., 2014).

bTB infection patterns in this study likely do not result purely from these resistance phenotypes and underlying immune mechanisms. Contact patterns and connectivity in other animal systems have been shown to influence disease exposure, incidence, and resulting spread (Jones, Betson, & Pfeiffer, 2017; Lange & Thulke, 2017; Rushmore et al., 2013). Coinfecting pathogens along with seasonal fission–fusion dynamics affecting contact patterns in African buffalo herds could also drive variable patterns in bTB spread in this system (Cross et al., 2004). We observed a significant influence of herd membership on variation in time to onset of bTB in the variance‐partitioning models, suggesting that exposure differences at a large geographic scale may be playing a role in onset of disease.

In conclusion, here, we provide evidence for multiple resistance phenotypes with different, context‐dependent fitness costs and benefits. Though environmental factors likely contribute to variation in time to infection and resulting pathology, we demonstrate that conversion age has a genetic basis and proliferation resistance associates with better general health. Furthermore, these distinct forms of bTB resistance exemplify “fast” and “slow” pace‐of‐life syndromes, providing a novel example of multiple life history strategies coexisting within a single wild mammal population. Future work involving genetic association and quantitative genetic modeling will help to pinpoint plausible mechanisms of resistance and project its evolutionary trajectory in this system.

## CONFLICT OF INTEREST

None declared.

## AUTHOR CONTRIBUTIONS

HFT conducted all molecular laboratory work, developed the heritability analysis workflow, conducted all statistical analyses, and wrote this manuscript. VOE and AEJ conceived the ideas for the main bTB project for which these data were primarily collected, designed the experiment, oversaw data collection, and obtained funding for the work. BRB, EEG, JMS, and RSS conducted field data collection and immunological laboratory work and harmonized project data. PEB completed all diagnostic histopathology to characterize the pathology of bTB infections and oversaw animal safety during field collections. EGH, NlR, and PDvH provided the *Syncerus caffer* genome and advice on early analyses. All authors contributed critically to manuscript drafts and gave final approval for publication.

## DATA ACCESSIBILITY

Fitness measures, demographic covariates, bTB status data, and SNP genotypes are available on Dryad https://doi.org/10.5061/dryad.s3b0n42.
