# Supplementary material for: Context‐dependent costs and benefits of tuberculosis resistance traits in a wild mammalian host
Source: Ecol Evol. 2018 Dec 6;8(24):12712–26. doi: 10.1002/ece3.4699 (PMC6308860; doi:10.1002/ece3.4699)
Supplement: Supplementary file 1 [file ECE3-8-12712-s001.docx]

**Table S1: AIC values for the infection resistance heritability model.**

| Model^b^ | AIC^a^ | ΔAIC­­­­­­­­ |
| --- | --- | --- |
| Time to onset of bTB | | |
| ~ Treatment + random([**r**]+[**e**]) | 3.34 | - |
| ~[Intercept] + random([**r**]+[**e**]) | 5.09 | 1.75 |
| ~ Final age + Treatment + random([**r**]+[**e**]) | 23.11 | 19.77 |
| ~ Final age + random([**r**]+[**e**]) | 24.44 | 21.10 |

^a^Akaike’s Information Criterion (AIC) values reported are based on a penalized log likelihood model fit. Models for time to onset of bTB are presented in order of increasing AIC. We selected the model with the lowest AIC value and checked residual plots (top model).

^b^The relatedness coefficient matrix and shared environment matrix are represented by [**r**] and [**e**], respectively, and were incorporated into each Cox mixed effects model as the correlation structure of the random effect (Animal ID).

**Table S2: AIC and marginal R^2^ values for models of fitness metrics among bTB resistance phenotypes.**

| Model^b^ | R^2^_marg_ | ­­­­­­­­ AIC^a^ | ΔAIC |
| --- | --- | --- | --- |
| Average Body Condition Before & After Infection | | | |
| ~ Time + Conversion Age + Pathology + Herd | 0.547 | 98.16 | - |
| ~ Time + Conversion Age + Pathology + Herd + Time*Conversion Age | 0.553 | 99.27 | 1.11 |
| ~ Time + Conversion Age + Pathology + Herd + Initial Age | 0.549 | 99.93 | 1.77 |
| ~ Time + Conversion Age + Pathology + Herd + Treatment | 0.548 | 100.06 | 1.90 |
| ~ Time + Conversion Age + Pathology + Herd + Conversion Age*Pathology | 0.547 | 100.13 | 1.97 |
| ~ Time + Conversion Age + Pathology + Herd + Time*Pathology | 0.547 | 100.16 | 2.00 |
| ~ Time + Conversion Age + Pathology + Herd + Initial Age + Time*Conversion Age | 0.555 | 101.05 | 2.89 |
| ~ Time + Conversion Age + Pathology + Treatment + Herd + Time*Conversion Age | 0.554 | 101.17 | 3.01 |
| ~ Time + Conversion Age + Pathology + Herd + Time*Conversion Age + Conversion Age*Pathology | 0.553 | 101.24 | 3.08 |
| ~ Time + Conversion Age + Pathology + Herd + Time*Conversion Age + Time*Pathology | 0.553 | 101.27 | 3.11 |
| ~ Time + Conversion Age + Herd | 0.504 | 101.69 | 3.53 |
| ~ Time + Conversion Age + Pathology + Treatment + Herd + Initial Age | 0.549 | 101.89 | 3.73 |
| ~ Time + Conversion Age + Herd + Time*Conversion Age | 0.510 | 102.80 | 4.64 |
| ~ Time + Conversion Age + Pathology + Treatment + Herd + Initial Age + Time*Conversion Age | 0.555 | 103.00 | 4.84 |
| ~ Time + Conversion Age + Pathology + Herd + Initial Age + Time*Conversion Age + Conversion Age*Pathology | 0.555 | 103.03 | 4.87 |
| ~ Time + Conversion Age + Pathology + Herd + Initial Age + Time*Conversion Age + Time*Pathology | 0.555 | 103.04 | 4.88 |
| ~ Time + Conversion Age + Pathology + Treatment + Herd + Time*Conversion Age + Conversion Age*Pathology | 0.554 | 103.10 | 4.94 |
| ~ Time + Conversion Age + Pathology + Treatment + Herd + Initial Age + Conversion Age*Pathology | 0.549 | 103.86 | 5.70 |
| ~ Time + Conversion Age + Herd + Initial Age + Time*Conversion Age | 0.512 | 104.59 | 6.43 |
| ~ Time + Conversion Age + Pathology + Treatment + Herd + Initial Age + Time*Conversion Age + Conversion Age*Pathology | 0.555 | 104.97 | 6.81 |
| ~ Time + Conversion Age + Pathology + Treatment + Herd + Initial Age + Time*Conversion Age + Time*Pathology | 0.555 | 105.00 | 6.84 |
| ~ Time + Conversion Age + Pathology + Herd + Initial Age + Time*Conversion Age + Time*Pathology + Conversion Age*Pathology | 0.555 | 105.03 | 6.87 |
| ~ Time + Conversion Age + Pathology + Treatment + Herd + Time*Conversion Age + Time*Pathology + Conversion Age*Pathology | 0.554 | 105.10 | 6.94 |
| ~ Time + Pathology + Herd | 0.472 | 105.34 | 7.18 |
| ~ Time + Conversion Age + Pathology + Treatment + Herd + Initial Age + Time*Pathology + Conversion Age*Pathology | 0.549 | 105.86 | 7.70 |
| ~ Time + Conversion Age + Treatment + Herd + Initial Age + Time*Conversion Age | 0.517 | 106.05 | 7.89 |
| ~ Time + Conversion Age + Pathology + Treatment + Herd + Initial Age + Time*Conversion Age + Time*Pathology + Conversion Age*Pathology | 0.555 | 106.97 | 8.81 |
| ~ Time + Conversion Age + Pathology | 0.406 | 111.73 | 13.57 |
| ~ Time + Conversion Age + Pathology + Time*Conversion Age | 0.412 | 112.84 | 14.68 |
| ~ Time + Conversion Age + Pathology + Initial Age + Time*Conversion Age | 0.413 | 114.77 | 16.61 |
| ~ Time + Conversion Age + Pathology + Treatment + Initial Age + Time*Conversion Age | 0.413 | 116.77 | 18.61 |
| ~ Time + Conversion Age + Pathology + Treatment + Initial Age + Time*Conversion Age + Conversion Age*Pathology | 0.426 | 117.59 | 19.43 |
| ~ Time + Conversion Age + Pathology + Treatment + Initial Age + Time*Conversion Age + Time*Pathology + Conversion Age*Pathology | 0.426 | 119.59 | 21.43 |
| ~ Conversion Age + Pathology + Herd | 0.291 | 125.54 | 27.38 |
|  |  |  |  |
| Average Reproduction Rate Before & After Infection^c^ | | | |
| ~ Time + Conversion Age + Pathology + Treatment + Herd | 0.545 | 51.01 | - |
| Model^b^ | R^2^_marg_ | AIC^a^ | ΔAIC |
| ~ Time + Conversion Age + Pathology + Herd | 0.528 | 51.44 | 0.43 |
| ~ Time + Conversion Age + Pathology + Treatment + Herd + Conversion Age*Pathology | 0.556 | 51.47 | 0.46 |
| ~ Time + Conversion Age + Pathology + Treatment + Herd + Time*Pathology | 0.555 | 51.58 | 0.57 |
| ~ Time + Conversion Age + Pathology + Treatment + Herd + Time*Conversion Age | 0.555 | 51.63 | 0.62 |
| ~ Time + Conversion Age + Pathology + Treatment + Herd + Time*Pathology + Conversion Age*Pathology | 0.567 | 51.99 | 0.98 |
| ~ Time + Conversion Age + Pathology + Treatment + Herd + Time*Conversion Age + Conversion Age*Pathology | 0.565 | 52.05 | 1.04 |
| ~ Time + Conversion Age + Pathology + Treatment + Herd + Time*Conversion Age + Time*Pathology | 0.564 | 52.30 | 1.29 |
| ~ Time + Conversion Age + Pathology + Treatment + Conversion Age*Pathology | 0.536 | 52.34 | 1.33 |
| ~ Time + Conversion Age + Pathology + Herd + Conversion Age*Pathology | 0.534 | 52.68 | 1.67 |
| ~ Time + Conversion Age + Pathology + Treatment + Herd + Time*Conversion Age + Time*Pathology + Conversion Age*Pathology | 0.574 | 52.69 | 1.68 |
| ~ Time + Conversion Age + Pathology + Treatment + Time*Pathology + Conversion Age*Pathology | 0.546 | 52.93 | 1.92 |
| ~ Time + Conversion Age + Pathology + Treatment + Initial Age + Herd | 0.546 | 52.96 | 1.95 |
| ~ Time + Conversion Age + Pathology + Herd + Time*Pathology + Conversion Age*Pathology | 0.544 | 53.28 | 2.27 |
| ~ Time + Conversion Age + Pathology + Treatment + Herd + Initial Age + Conversion Age*Pathology | 0.556 | 53.47 | 2.46 |
| ~ Time + Conversion Age + Pathology + Time*Conversion Age + Time*Pathology + Conversion Age*Pathology | 0.525 | 53.68 | 2.67 |
| ~ Time + Conversion Age + Pathology + Treatment + Herd + Initial Age + Time*Pathology + Conversion Age*Pathology | 0.566 | 53.99 | 2.98 |
| ~ Time + Conversion Age + Pathology + Herd + Time*Conversion Age + Time*Pathology + Conversion Age*Pathology | 0.552 | 54.04 | 3.03 |
| ~ Time + Conversion Age + Pathology + Treatment + Herd + Initial Age + Time*Conversion Age + Conversion Age*Pathology | 0.565 | 54.05 | 3.04 |
| ~ Time + Conversion Age + Pathology + Treatment | 0.508 | 54.13 | 3.12 |
| ~ Time + Conversion Age + Pathology + Treatment + Herd + Initial Age + Time*Conversion Age + Time*Pathology | 0.564 | 54.25 | 3.24 |
| ~ Time + Conversion Age + Pathology + Treatment + Herd + Initial Age + Time*Conversion Age + Time*Pathology + Conversion Age*Pathology | 0.574 | 54.69 | 3.68 |
| ~ Time + Conversion Age + Pathology + Treatment + Initial Age + Time*Conversion Age + Time*Pathology + Conversion Age*Pathology | 0.555 | 55.66 | 4.65 |
| ~ Time + Conversion Age + Pathology + Herd + Initial Age + Time*Conversion Age + Time*Pathology + Conversion Age*Pathology | 0.554 | 55.74 | 4.73 |
| ~ Time + Conversion Age + Treatment + Herd | 0.492 | 56.31 | 5.30 |
| ~ Conversion Age + Pathology + Treatment + Herd | 0.374 | 69.94 | 18.93 |
| ~ Conversion Age + Pathology + Treatment + Herd + Conversion Age*Pathology | 0.385 | 70.82 | 19.81 |
| ~ Time + Pathology + Treatment + Herd | 0.251 | 80.52 | 29.51 |

^a^Akaike’s Information Criterion (AIC) values reported are based on a log likelihood model fit. Models for each fitness metric are presented in order of increasing AIC. We considered AIC value, marginal R^2^, and checked residual plots when selecting the final model (top model).

^b^ Mixed effects models fit by maximum likelihood included ‘Animal ID’ as a random effect, as each fitness metric was assessed before and after bTB infection (Time). ‘Conversion age’ is the age at which an animal became bTB positive and serves as the continuous metric of infection resistance in these models. ‘Pathology’ is each animal’s residual value for the lung lesions over time with bTB nonlinear regression and serves as the continuous metric of proliferation resistance in these models. Body condition was assessed on a five point scale common in livestock. Reproductive rate was calculated as calves per year during the study period.

^c^Models for reproduction rate also include an offset term of years in study to account for differences in observation times.

**Table S3: AIC and R^2^ values for models of survival following bTB infection.**

| Model^b^ | R^2^ | AIC^a^ | ΔAIC­­­­­­­­ |
| --- | --- | --- | --- |
| Death Risk | | | |
| ~ Conversion Age+ Treatment + Herd | 0.176 | 63.81 | - |
| ~ Treatment + Herd | 0.137 | 64.41 | 0.60 |
| ~ Conversion Age+ Herd | 0.061 | 65.15 | 1.34 |
| ~ Conversion Age+ Treatment | 0.124 | 65.27 | 1.46 |

^a^Akaike’s Information Criterion (AIC) values reported are based on a log likelihood model fit. Models for each fitness metric are presented in order of increasing AIC. We considered AIC value, marginal R^2^, and checked residual plots when selecting the final model (top model).

^b^Cox Proportional Hazards models describing death risk once infected in bTB converted animals (n=56, deaths=10). Herd and anti-helminthic bolus (treatment) have been previously shown to impact survival time with bTB (45) and are part of the selected model in this subset of animals as well.
